# The role of first-trimester systemic immune-inflammation index for the prediction of gestational diabetes mellitus

**DOI:** 10.1590/1806-9282.20240532

**Published:** 2024-09-30

**Authors:** Nizamettin Bozbay, Anara Medinaeva, Fikret Akyürek, Gokcen Orgul

**Affiliations:** 1 Selcuk University, Faculty of Medicine, Department of Perinatology – Konya, Turkey.; 2 Selcuk University, Faculty of Medicine, Department of Obstetrics and Gynecology – Konya, Turkey.; 3 Selcuk University, Faculty of Medicine, Department of Biochemistry – Konya, Turkey.

**Keywords:** First trimester, GDM, Inflammation, LMR, SI

## Abstract

**OBJECTIVE:**

The aim of this study was to investigate the role of systemic immune-inflammation index, neutrophil–lymphocyte ratio, lymphocyte–monocyte ratio, and platelet–lymphocyte ratios calculated in the first trimester as inflammatory markers in predicting gestational diabetes mellitus diagnosis.

**METHODS:**

This study was conducted retrospectively at a tertiary center between January 2020 and June 2023. A total of 111 pregnant women with gestational diabetes and 378 pregnant women in the control group were included in the study. Systemic immune-inflammation index, neutrophil–lymphocyte ratio, lymphocyte–monocyte ratio, and platelet–lymphocyte ratios values were compared between the gestational diabetes mellitus group patients and the healthy group. Receiver operating characteristic analysis curve was used for predicting gestational diabetes mellitus using systemic immune-inflammation index and lymphocyte–monocyte ratio.

**RESULTS:**

In pregnant women in the first trimester, systemic immune-inflammation index and lymphocyte–monocyte ratio values based on routine complete blood count parameters were found to be statistically significantly higher in gestational diabetes mellitus patients compared to healthy patients, while neutrophil–lymphocyte ratio and platelet–lymphocyte ratios values were found to be similar (p=0.033, p=0.005, p=0.211, and p=0.989). For predicting gestational diabetes mellitus, a cut-off value of 655.75 for systemic immune-inflammation index resulted in 80.2% sensitivity and 34.4% specificity, and a cut-off value of 3.62 for lymphocyte–monocyte ratio resulted in 56.8% sensitivity and 63.2% specificity, indicating good discriminatory ability.

**CONCLUSION:**

We believe that systemic immune-inflammation index and lymphocyte–monocyte ratio values measured in the first-trimester complete blood count parameters are effective in predicting gestational diabetes mellitus but are not effective in determining insulin requirement.

## INTRODUCTION

Gestational diabetes mellitus (GDM) is characterized by increased secretion of diabetogenic hormones from the placenta and an inadequate response of the pancreas to insulin resistance^1^. GDM, which is defined as a state of hyperglycemia that is first recognized during pregnancy, is currently the most common medical complication in pregnancy. According to the latest estimates of the International Diabetes Federation, GDM affects approximately 14.0% (95% confidence interval: 13.97–14.04%) of pregnancies worldwide, representing approximately 20 million births annually^2^. Elevated glucose levels can lead to maternal and fetal complications. Mothers with GDM are at risk of developing gestational hypertension, pre-eclampsia, and termination of pregnancy via cesarean section^3^. In addition, GDM increases the risk of complications, including cardiovascular disease, obesity, and impaired carbohydrate metabolism, leading to the development of type 2 diabetes in both mothers and infants^4^.

Early diagnosis and appropriate treatment can reduce the risk of obstetric complications. It is recommended that all pregnant women undergo GDM screening with an oral glucose tolerance test (OGTT) between 24 and 28 weeks of gestation. Screening in the first trimester is even recommended for high-risk pregnancies^5^.

Several factors play a role in the etiopathogenesis of diabetes. Its development is primarily caused by a combination of two main factors: defective insulin secretion by pancreatic β-cells and the inability of insulin-sensitive tissues to respond to insulin^6^. Recently, there has been increased interest in the role of inflammation in the causes of diabetes. Inflammation leads to an increase in immune cells, including M1 macrophages and T lymphocytes, which secrete proinflammatory cytokines that induce insulin resistance^7^.

Inflammation is a biological response of the immune system that can be triggered by a variety of factors, including pathogens, damaged cells, and toxic compounds. These factors may induce acute and/or chronic inflammatory responses in the heart, pancreas, liver, kidney, lung, brain, intestinal tract, and reproductive system, potentially leading to tissue damage or disease^8^. Systemic inflammation and immune status are effective determinants based on complete blood count (CBC) parameters, including neutrophil–lymphocyte ratio (NLR), lymphocyte–monocyte ratio (LMR), and platelet–lymphocyte ratio (PLR). A new index known as the systemic immune-inflammation index (SII) is calculated using peripheral platelet, neutrophil, and lymphocyte counts. The use of these parameters in pregnancy is increasingly being recognized, as they can be easily calculated.

Based on previous research emphasizing the role of inflammatory blood parameters in GDM, we aimed to investigate whether calculating SII, NLR, LMR, and PLR markers from CBC counts in the first trimester would be effective in predicting GDM together with insulin requirement.

## METHODS

This study was conducted at the Department of Obstetrics and Gynecology, Faculty of Medicine, Selçuk University, between January 1, 2020, and June 30, 2023. Patient data were obtained retrospectively from file archives and electronic records. The study was planned in accordance with the principles of the Helsinki Declaration, and approval was obtained from the Local Ethics Committee of Selçuk University Faculty of Medicine (Date: 26.09.2023 and Ethics Committee number: 2023/455) before starting the study.

During the study period, a total of 111 cases were included in the patient group, who were diagnosed and treated for GDM in our hospital, and all deliveries occurred in our hospital. According to the criteria of the American College of Obstetricians and Gynecologists (ACOG), these cases were divided into two groups: gestational diabetes patients who were managed without medication were included in the GDM A1 group and gestational diabetes patients requiring medication (insulin) to achieve glycemic control were included in the GDM A2 group^9^. A control group was selected at a ratio of 1:3 based on the number of patients. A total of 378 healthy pregnant women who gave birth in our hospital and had no additional diseases were randomly selected and included in the control group. The study included healthy singleton pregnancies diagnosed with GDM based on OGTT results. Exclusion criteria included pregestational diabetes (type 1 and type 2 DM), women with twin gestation, fetal structural or chromosomal anomalies, comorbidities, and obstetric complications (preeclampsia, placenta previa, PPROM, IUGR, etc.).

In our country, CBC in the first trimester is routinely recommended for all pregnant women. Initially, CBC results of all cases including white blood cell count (WBC), hemoglobin (Hb), hematocrit (Hct), red blood cell count (RBC), platelet count (PLT), neutrophil count (NEU), monocyte count (MON), lymphocyte count (LYM), mean platelet volume (MPV), platelet distribution width (PDW), red cell distribution width (RDW), eosinophil count (EOZ), and basophil count (BAZ) were obtained. Then, the formulation for NLR was calculated as the ratio of neutrophil count to lymphocyte count; the formulation for LMR was calculated as the ratio of lymphocyte count to monocyte count, and the formulation for PLR was calculated as the ratio of platelet count to lymphocyte count. Additionally, the SII was calculated using the formulation of platelet count multiplied by neutrophil count divided by lymphocyte count.

### Statistical analysis

The analyses were conducted using SPSS (Statistical Package for Social Sciences; SPSS Inc., Chicago, IL) version 22. Descriptive data were presented as n, % for categorical variables, and mean±standard deviation (mean±SD) or median with interquartile range (25–75 percentile values) for continuous variables. Pearson’s chi-square test (χ^2^) was applied for the comparison of categorical variables between groups. The normal distribution of continuous variables was evaluated using the Kolmogorov-Smirnov test. The Mann-Whitney U-test was used for comparing two groups, and the Kruskal-Wallis test was used for comparing more than two groups for continuous variables. Receiver operating characteristic (ROC) curves were drawn to measure the value of various parameters in predicting the presence of GDM. A significance level of p<0.05 was considered statistically significant in the analyses.

## RESULTS

A total of 489 patients were included in the study, comprising the GDM A1 group (n=50), the GDM A2 group (n=61), and the control group (n=378). When comparing the general characteristics and obstetric histories of the groups, no statistically significant differences were found between the groups (Table 1).

**Table 1:** Comparison of general characteristics and obstetric histories between groups.

	GDMA 1 (n=50)	GDMA 2 (n=61)	Control groups (n=378)	p-value*
	Median (min–max)	Median (min–max)	Median (min–max)	
Age	33.5 (21–44)	33 (24–45)	32 (19–44)	0.156
Gravidity	4 (1–6)	3 (1–4)	3 (1–5)	0.234
Parity	1 (0–5)	1 (0–4)	1 (0–4)	0.124
Weight (kg)	73 (51–118)	76 (51–110)	72 (44–122)	0.060
Height (cm)	160 (150–170)	161 (150–168)	160 (148–175)	0,376
BMI (kg/m^2^)	28.8 (20.7–43.3)	31.6 (20.7–38.4)	28 (18.4–34.9)	0.090

*The Kruskal-Wallis test was applied, kg: kilograms, cm: centimeters, kg/m^2^: kilograms per square meter, min: minimum, max: maximum.

The data obtained after the comparison of inflammatory indices among the three groups are shown in Table 2. In the GDM group patients, SII, NLR, LMR, and PLR values were found to be higher compared to the control group. However, only SII (p=0.033) and LMR (p=0.001) values were statistically significantly greater than those of healthy individuals.

**Table 2:** Comparison of systemic immune-inflammation index, neutrophil–lymphocyte ratio, lymphocyte–monocyte ratio, and platelet–lymphocyte ratios parameters among the groups.

	GDMA 1 (n=50)	GDMA 2 (n=61)	Control groups (n=378)	p-value*
	Median (min–max)	Median (min–max)	Median (min–max)	
SII	876.5 (306.1–1691.6)^a^	894.9 (374.0–2652.0)^a^	776,1 (224.0–1465.0)^b^	**0.033**
NLR	3.1 (1.3–5.8)	3.2 (1.3–6.4)	2.9 (0.4–4.77)	0.211
LMR	3.6 (1.6–5.5)^a^	3.9 (1.8–6.7)^a^	3,1 (1.4–4.9)^b^	**0.005**
PLR	132.8 (79.9–417.8)	134.8 (69.5–315.7)	131.6 (67.1–339.3)	0.989

*Kruskal-Wallis test was applied.

^a,b^The source of the difference. SII: systemic immune-inflammation index, NLR: neutrophil–lymphocyte ratio, LMR: lymphocyte–monocyte ratio, PLR: platelet–lymphocyte ratios. Bold: p<0.05 was considered significant.

**Figure 1:**
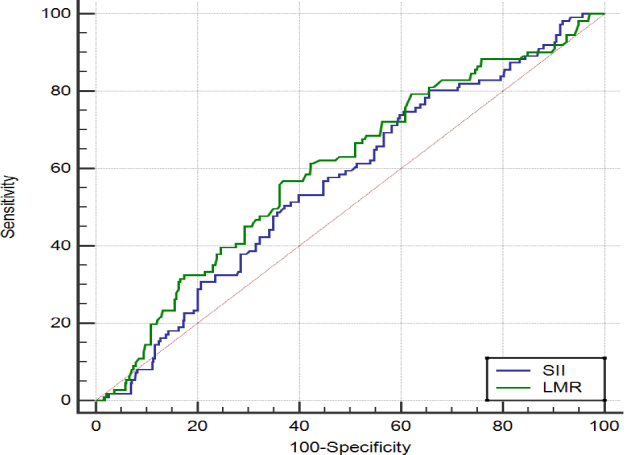
Receiver operating characteristic curve of systemic immune-inflammation index and lymphocyte–monocyte ratio ratio for the presence of gestational diabetes mellitus.

The ROC curve graph for the use of SII and LMR in GDM prediction is shown in Figure 1. When the value of 655.75 is taken as the cut-off for SII, a sensitivity of 80.2% and a specificity of 34.4% are determined. For LMR, when the value of 3.62 is taken as the cut-off, a sensitivity of 56.8% and a specificity of 63.2% are determined.

## DISCUSSION

Gestational diabetes mellitus (GDM) is associated with significant maternal and neonatal morbidity and mortality^10^. However, managing GDM appropriately can reduce adverse perinatal outcomes^11^. Lifestyle changes such as implementing a diabetic diet for normal blood glucose levels, reducing extra calories, and increasing physical activity may be possible^12^. Additionally, close antenatal monitoring, screening for fetal well-being, and maternal insulin therapy when necessary reduce obstetric complications associated with GDM^13^.

Many international guidelines, such as those of the American Diabetes Association and the World Health Organization, recommend early antenatal testing and diagnosis of diabetes during pregnancy for women at risk of diabetes^14^. Therefore, routine screening for GDM in early pregnancy is important. In our study, due to the relationship between GDM and inflammation, we aimed to predict GDM using CBC, a simple blood test parameter routinely examined in early pregnancy. We found that inflammatory markers in the first trimester were higher in cases of developing diabetes compared to healthy pregnancies. While statistically significant differences were found for SII and LMR values among the four markers investigated, although NLR and PLO values were not statistically significant, they were found to be elevated. Although SII and LMR inflammatory indices were found to be useful in predicting GDM, their use in determining insulin requirements was found to be inadequate.

Recent studies underline that the adverse pregnancy outcomes in GDM are not solely attributed to specific causes such as increased insulin resistance and glucose intolerance. It has been found that the mother’s immune system causes adverse pregnancy outcomes by disrupting metabolic pathways in the placenta, fetal growth, and fetal neurodevelopment^15^. Another study in 2021 showed that genetic differences (FABP4, DKK1, CXCL10, and IL1RL1) play a potential role in the development of GDM by leading to placental inflammation^16^. In one study, serum cartonectin distributions were investigated in pregnant women diagnosed with GDM. The study and control groups were similar in terms of median serum cartonectin concentrations (6.28 ng/mL and 7.13 ng/mL, respectively, p=0.165)^17^.

Inflammation causes cellular damage through various mechanisms and plays a role in the pathogenesis of various diseases. GDM, one of the diseases caused by inflammation, has been found to develop due to an imbalance in the release ratio of proinflammatory and anti-inflammatory cytokines, resulting in insulin resistance^18^. In a study comparing pregnant women with hyperglycemia with healthy pregnant women, high NLR and MLR values were found to be associated with adverse pregnancy and perinatal outcomes^19^. Beser et al. investigated NLR, PLR, LMR, and SII values in hospitalized patients to predict the severity of hyperemesis gravidarum (HEG). Low specificity and sensitivity were found in predicting severe HEG patients^20^.

The SII, calculated using counts of platelets, neutrophils, and lymphocytes from peripheral blood cells, is recently being investigated in GDM. In a study by Ergani et al., the inflammatory marker SII was found to be significantly higher in the third trimester than in the first trimester and was associated with increased amniotic fluid with high OGTT^21^. In our study, SII was found to be significantly higher in cases of developing GDM compared to healthy pregnancies in the early stages of pregnancy.

The LMR is calculated by dividing the absolute lymphocyte count by the absolute monocyte count. Generally, there is a decrease in the LMR compared to non-pregnant individuals during pregnancy. This change is part of the body’s adaptations to support pregnancy and prevent the immune-mediated rejection of the fetus. However, individual differences may occur^22^. There is no previous study investigating the change in LMR index in GDM patients. Since this study is the first of its kind, we believe it will contribute to the literature.

There are some limitations in our study. The retrospective design, single-center analysis, and limited number of patients are important limitations of our study. We believe that more extensive supportive studies are needed for this study.

Although there are several causal risk factors in the development of gestational diabetes, its etiology is not yet fully elucidated. Our knowledge about the clinical significance of inflammatory markers in predicting GDM is limited. In this study, we investigated the role of inflammation in predicting GDM in the first trimester. There are insufficient studies evaluating SII, NLR, PLR, and LMR parameters in predicting GDM in the literature. We believe that this study has the potential to support clinicians in routine clinical practice. We found that the SII and LMR values measured in the first-trimester CBC parameters were effective in predicting GDM, although they were not effective in predicting insulin requirements among GDM groups. Larger studies aiming to develop a diagnostic algorithm using SII and LMR values in addition to maternal characteristics and other GDM risk factors are needed.
